# A Non Platinum Regimen for the Treatment of Recurrent or Metastatic Squamous Cell Carcinoma of the Head and Neck Region. Results From an Extended Phase II Study With Paclitaxel and Capecitabine

**DOI:** 10.3389/fonc.2018.00243

**Published:** 2018-06-29

**Authors:** Jens K. D. Bentzen, Claus Andrup Kristensen, Marie Overgaard, Carsten Rytter, Kenneth Jensen, Hanne Sand Hansen

**Affiliations:** ^1^Department of Oncology, Herlev Hospital, Herlev, Denmark; ^2^Department of Oncology, The Finsen Centre, Rigshospitalet, Copenhagen, Denmark; ^3^Department of Oncology, Aarhus University Hospital, Aarhus, Denmark

**Keywords:** paclitaxel, capecitabine, phase II study, head and neck squamous cell carcinoma, recurrence, toxicity, non platinum

## Abstract

**Background:**

This study presents the results of an extended phase II study originally published in 2007, regarding the antitumor activity and toxicity of a non-platinum containing regimen with paclitaxel and capecitabine for the treatment of recurrent or disseminated squamous cell carcinoma of the head and neck region. Fifty patients were included in the original study.

**Materials and methods:**

A total of 183 patients with recurrent or disseminated squamous cell carcinoma were eventually included in the extended study. There were 37 women and 146 men. The mean age was 56 years. Performance status (WHO) was as follows: WHO 0:31, WHO 1:107, and WHO 2:45 patients. The treatment consisted of paclitaxel 175 mg/m^2^, once every third week and capecitabine 825 mg/m^2^ p.o. b.i.d for 2 weeks.

**Results:**

The overall response rate (complete response and partial response) according to the WHO criteria was: 33% (CI 26–40). The median progression-free survival was 4.8 (CI 4.2–5.4) months. The median overall survival (OS) was 8.9 (CI 7.6–9.5) months. Compliance was good. Of the 1,131 cycles, only 13% had to be administered with a reduced dose and/or postponed to a later date. Toxicity was mild and grades 3 and 4 toxicities were uncommon. Two toxic deaths were registered though.

**Conclusion:**

The response rate and the OS for this low toxicity regimen makes it a feasible alternative for not cisplatin eligible patients.

## Introduction

In 2007, we published the results of a phase 2 study with paclitaxel and capecitabine for the treatment of recurrent or metastatic squamous cell carcinoma of the head and neck region (1). Fifty patients were included in the study. The aim of the study was to determine the efficacy and toxicity of the regimen, which we hoped could turn out to be an alternative to the widely accepted, but rather toxic, 5 Flourouracile (5 FU) and cisplatin regimen. The overall response rate [partial response (PR) and complete response (CR)] according to the WHO criteria was 42%, the median overall survival (OS) was 8 months, toxicity was moderate, and the patient compliance was very good. The results of this treatment regimen compared favorably with published data for the cisplatin and 5 FU regimen. As we did not find it likely that we would be able to include enough patients to power a phase 3 study, we decided to continue to accrue more patients to provide a more robust estimate of the response rate and survival. It is worth noting that patients with a WHO performance status of 2 were eligible for the study (by contrast to most other phase 2 studies). We believed the regimen would only be mildly toxic and, therefore, potentially beneficial also for performance level 2 patients. The primary outcome of the study was RR and toxicity, and secondary outcome was OS and compliance. In this paper, we will also report progression-free survival (PFS) as secondary outcome. An additional 133 patients were accrued so that the extended study ended up with 183 patients in total. This paper presents the final results of the extended study.

## Materials and Methods

### Patient Eligibility

Patients were eligible if they had histologically confirmed recurrent or metastatic squamous cell carcinoma of the head and neck region, not suitable for curative radiotherapy or salvage surgery (all patients with recurrences were evaluated at a multidisciplinary tumor board before they were informed about the study). They should have measurable disease in minimum two dimensions on Ultrasound, MRI-scan, or CT-scan; age between 18 and 75 years and a WHO performance status less than or equal to two; no previous chemotherapy for at least 1 month; no other severe life shortening disease or other malignant disease and adequate bone marrow, liver, and renal functions. They had to be mentally well and psychologically capable of understanding and adhering to the treatment plan and all patients had to sign an informed consent form. Lesions were measured at baseline and after every three series (every ninth week). Toxicity was measured by blood samples and patient interview after every treatment.

The study was conducted according to the Helsinki Declaration II, and the protocol was approved by the Ethics committees of Copenhagen Denmark.

### Treatment

Day 1: Paclitaxel 175 mg/m^2^ i.v. over 3 h. Days 1–14: Capecitabine 825 mg/m^2^/dose orally b.i.d., with 200 ml water taken less than 30 min after a meal. After a 1 week interval without medication, the treatment was repeated. The patients received the following i.v. premedication 30 min prior to administration of paclitaxel: Dexamethazone 10 mg, Clemastin (Tavegyl^®^) 2 mg, Nizatidin (Nizax^®^) 100 mg. For details about paclitaxel and capecitabine, we kindly refer to the previously published paper regarding the first 50 patients (1).

### Statistics

All statistical analysis was based on the intention to treat principle. One patient did not receive any treatment and was thus not eligible for the toxicity analysis, but was included in the survival estimates, using the enrollment date as first date. The criteria for response and toxicity were based on the standard definition of WHO (2, 3). PFS was defined as the time from the first day of chemotherapy to progression or death. Survival curves were generated according to the method of Kaplan and Meier (4). Survival time was calculated from the first day of chemotherapy until death. Surviving patients were censored at the day of their last follow-up visit. For the statistical analyses, we used software from SPSS statistics version 17.0 and Graph Pad Prism 5.0.

## Results

### Patient Characteristics

A total of 183 patients from three centers were recruited to the study. There were 37 women and 146 men. The median age was 56 years (range 23–75 years). One patient never received any treatment and was thus not eligible for the toxicity analysis. The patients were enrolled between 2000 and 2005. The basic characteristics of the patients are listed in Table 1, and the primary TNM status is shown in Table 2. Most patients had been treated with radiotherapy as their primary treatment, which reflects the primary treatment strategy in Denmark for head and neck cancer at that time. No patients had received chemotherapy as part of their curative treatment and, as it turned out, none of the patients had previous systemic treatment for their recurrences before entering the study. About two-third of the patients had loco regionally recurrences only and about one-third had metastatic disease. Forty-five patients (25%) had a performance status of 2. Being an old study, none of the oropharyngeal cancer patients were tested for HPV status.

**Table 1:** Baseline characteristics of the patients.

Variable	Number of patients	%
Gender		
Female	37	20.2
Male	146	79.8
Age median	56 (range 23–75)	
**Primary tumor site**		
Nasopharynx	15	8.2
Oropharynx	53	28.8
Hypopharynx	25	13.6
Larynx	27	14.7
Oral cavity	45	24.5
Other	18	9.7
**Primary treatment**		
Radiotherapy only	120	65.2
Surgery only	9	4.9
Radiotherapy and surgery	49	26.7
No primary treatment	2	1.1
Chemotherapy	0	0
Other	4	2.2
**Extent of disease at enrollment**		
Local only (*T*)	58	32
Regional only (*N*)	2	1
Distant only (*M*)	1	0.5
Local and regional	58	32
Local and distant	30	16
Regional and distant	1	0.5
Local, regional, and distant	33	18
**WHO performance status**		
0	31	16.8
1	107	58.7
2	45	24.5

**Table 2:** Initial TNM staging.

	N0	N1	N2	N3	Total
T0	2	0	2	0	4
T1	14	0	8	4	26
T2	25	14	22	11	72
T3	16	8	16	2	42
T4	16	6	11	4	37
Tx	0	2	0	0	2

Total	73	30	59	21	183

*Six patients had metastatic disease*.

### Response and Survival

**Figure 1:**
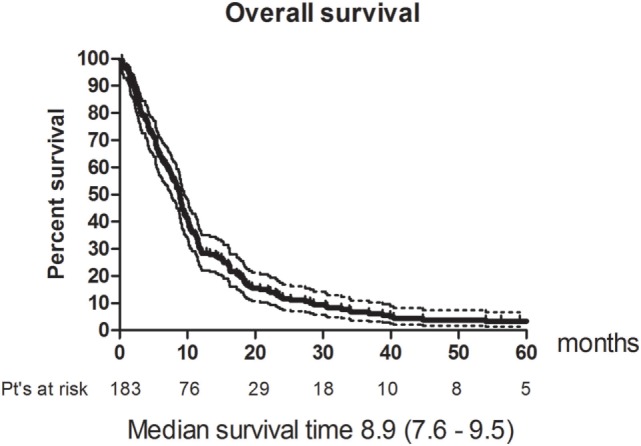
Overall survival for the study population. *N* = 183.

**Figure 2:**
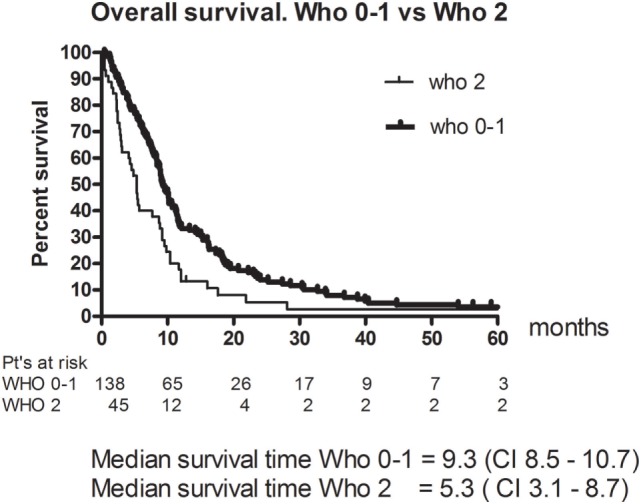
Overall survival for patients in WHO performance level 2 vs patients in level 0–1.

Eleven patients (6%) had a CR and 49 (26.8%) had a PR. The RR thus 33% (95% CI 26–40). The best response was no change (NC) for 67 (36.6%). 38 (20.8%) patients had progressive disease (PD) and 18 (9.8%) were not evaluable (NE) (Table 3). The median PFS was 4.8 months (95% CI 4.2–5.4). Figure 1 shows OS for all patients. The median OS was 8.9 months. However, as illustrated in Figure 2, the 45 of the patients, who had a WHO performance status of 2, did significantly worse than the rest. Median OS was only 5.3 months (95% CI 3.1–8.7) for this group compared with 9.3 months (95% CI 8.5–10.7) for the group of patients having performance level 0 or 1. The difference is highly significant (Log rank: *p* = 0.002). Among the patients with performance status 2, 24% had a PR or CR compared with 36% among patients with performance status 0 or 1.

**Table 3:** Best response: WHO criteria.

Response	Number of patients	%
Complete response	11	6
Partial response	49	26.8
NC	67	36.6
Progressive disease	38	20.8
Not evaluable	18	9.8

Total	183	100.0

### Toxicity

A total of 1,131 cycles of paclitaxel and capecitabine were delivered. The median number of cycles per patient being 6 (range 1–20). A dose reduction was necessary in 102 cycles (9%) and 87 (8%) of the cycles were postponed for a week or more. 151 (13%) cycles were either reduced or postponed. Table 4 summarizes the worst toxicity observed for each toxicity in a patient. Hair loss was expected and seen for most patients. Hand and foot syndrome and arthralgia/muscle pain were common side effects, but rarely exceeded grade 2. Hematologic toxicity was low as only 22 patients (12.1%) had grade 3 neutropenia (0.5–0.9 × 1,000/mm^3^) and none grade 4. There were 21 (11.5%) grade 3 infections (major infections without hypotension according to the WHO criteria) and two toxic deaths probably due to gastrointestinal infections. Both patients died after two series. One patient had up to 15 daily diarrheas. He was taken dehydrated to intensive care, where he died a few days after. The precise cause of his death remains unclear. The other patient was suspected for a gastrointestinal perforation and had an acute laparoscopy. No perforation was found. The patient deteriorated fast and died soon after. None of the patients had autopsies done.

**Table 4:** Worst toxicity (*N* = 182 patients. One patient did not receive any treatment).

Worst toxicity observed (WHO criteria)						

No. patients by grade (%)						

	N/A^a^	Grade 0	Grade 1	Grade 2	Grade 3	Grade 4
Skin (hand and foot syndrome)	15 (8.2)	77 (42.3)	49 (26.9)	25 (13.7)	16 (8.8)	0 (0)
Allergy	22 (12.1)	141 (77.5)	12 (6.6)	6 (3.3)	1 (0.5)	0 (0)
Mucositis	12 (6.6)	117 (64.3)	29 (15.9)	20 (11.0)	4 (2.2)	0 (0)
Heart	28 (15.4)	144 (79.1)	6 (3.3)	3 (1.6)	1 (0.5)	0 (0)
Diarrhea	12 (6.6)	0 (0)	84 (46.2)	49 (26.9)	28 (15.4)	0 (0)
Nausea	13 (7.1)	84 (46.2)	65 (35.7)	17 (9.3)	3 (1.6)	0 (0)
Vomiting	11 (6.0)	122 (67.0)	34 (18.7)	12 (6.6)	3 (1.6)	0 (0)
Uro-genital	24 (13.2)	144 (79.1)	3 (1.6)	9 (4.9)	2 (1.19)	0 (0)
Hair loss	12 (6.6)	10 (5.5)	28 (15.4)	69 (37.9)	63 (34.6)	0 (0)
Arthralgia/muscle pain	15 (8.2)	77 (42.3)	61 (33.5)	28 (15.4)	1 (0.5)	0 (0)
Neurotoxicity	16 (8.8)	84 (46.2)	48 (26.4)	22 (12.1)	12 (6.6)	0 (0)
Infection	8 (4.4)	91 (50.0)	30 (16.5)	30 (16.5)	21 (11.5)	2 (1.1)
Red blood cells	3 (1.6)	97 (53.3)	54 (29.7)	27 (14.8)	1 (0.5)	0 (0)
Neutrophils	4 (2.2)	118 (64.8)	22 (12.1)	16 (8.8)	22 (12.1)	0 (0)
Leukocytes	3 (1.6)	122 (67.0)	26 (14.3)	20 (11.0)	11 (6.0)	0 (0)
Thrombocytes	4 (2.2)	170 (93.4)	8 (4.4)	0 (0)	0 (0)	0 (0)

*^a^N/A or not applicable means that data are missing from the original patient records*.

## Discussion

The aim of this study was to get a more robust estimate of the efficacy and toxicity of the combination of paclitaxel and capecitabine since we thought that it could be a less toxic alternative to the standard combination of cisplatin and 5 FU for recurrent and/or metastatic head and neck cancer, and since we did not find it likely that a phase III study comparing the two regimes could be done. In this extended phase 2 study, the response rate was 33%, PFS was 4.8 months, and the median OS was 8.9 months. This corresponds well to earlier reported results with cisplatinum and 5 FU (5, 6). Both studies report RR of 32% for the cisplatin and 5 FU combination. The toxicity was mild, with hand and food syndrome being the most prominent finding. Compliance was good and the regimen could easily be given to patients on an outpatient basis. They only needed to come to the hospital once every third week for about 3–4 h to get their treatment. Patients with performance 2 were allowed entry to the study, as we believed the drug combination was only mildly toxic. However, the 45 patients with performance level 2 did significantly worse than the rest. Their median OS time was 5.3 months, which is not different from what could be expected without treatment (7), and as the study did not include any measures to provide QoL data, it is thus questionable whether they benefited from the treatment at all. Patients who have performance status 2 are seldom eligible for clinical trials for metastatic or recurrent SCCHN. In a phase III study by Argiris et al. (8), patients with PS 2 were also eligible if previously untreated for recurrent disease. The outcome for these patients remained, however, poor. This study uses the WHO criteria for reporting response and toxicity as this was the common criteria when this rather old study was initiated and can, therefore, not be directly compared to modern studies where one would use RECIST 1.1 for response and CTC for toxicity. The two studies to which we refer the response rates (5, 6) did also use WHO criteria for response. The CTC criteria are in general more comprehensive than the WHO criteria, but for the most prominent toxicity in this study, namely hand and foot syndrome, the scoring criteria are very similar in WHO and CTC. In 2008, the results from the EXTREME study were published (9). This phase 3 study showed superiority of the study combination cisplatin (or carboplatin), 5 FU and cetuximab vs the standard combination of cisplatin and 5 FU. The OS was 10.1 months vs 7.4 months. As this study until very recently has provided the only level 1 evidence for the treatment of recurrent and/or metastatic head and neck cancer, it has become the golden standard of treatment to these patients. It is a toxic regime though (10–12) and it demands that the patients are complying well since they have to come to the hospital on a weekly basis and they must either be hospitalized for 4–5 days during the treatment, or be able to cope with the pump at home for the duration of the 5 FU infusions. For fragile patients and platinum-resistant patients, it has been common practice to offer single agent treatment (13). The best documented being methotrexate, which in randomized trials have demonstrated OS in the order of 6 months (13). Other popular agents are cetuximab, docetaxel, and paclitaxel, which all have moderate toxicities. No single drug has proved to be superior to MTX in randomized studies until the recent phase III study by Ferris (14) with the checkpoint inhibitor Nivolumab vs doctors choice (MTX, cetuximab, or docetaxel) where a significant difference in OS was found [7.5 (95% CI 5.5–9.1) months vs 5.1 month]. Nivulomab was also less toxic than standard therapy (13% gr 3 and 4 adverse events vs 35%). Based on this study, Nivulomab has now been approved by the FDA and the EMA as second line treatment to platinum-resistant recurrent or metastatic SCCHN. Our study has OS in the same order as the Nivulomab arm, but with better RR and worse toxicity. Taxanes in general have been studied in several settings with head and neck cancer and have proved to have significant activity (15, 16), but they have never been compared to standard of care in phase 3 studies for recurrent or metastatic SCCHN. TPF vs PF has, however, been compared as induction chemotherapy for inoperable locoregionally disease (17) where TPF proved to be significantly superior. It is not very likely that we will see many phase 3 studies with taxanes in the near future, since at present, all focus seems to be on the possible role of immune checkpoint inhibitors. Out of 45 recently initiated studies with immune checkpoint inhibitors for head and neck cancer, only one (a phase I/II study) includes a taxane (18). The paclitaxel and capecitabine regimen in this study has for years been the most used treatment in Denmark to recurrent or metastatic head and neck cancer either, as first line treatment for fragile patients or as second line treatment to platinum resistant disease. The low toxicity profile of this drug combination could render it a suitable candidate to combine with other drugs. As the combination of paclitaxel and cetuximab seems to exert synergistic effect in the preclinical setting (19) (as is the case with cisplatin and cetuximab), the DAHANCA group has initiated a randomized phase 2 study (the DAHANCA 26 study) comparing the paclitaxel and capecitabine regimen presented in this paper to the same drugs in combination with weekly cetuximab.

## Datasets are Available upon Request

The raw data supporting the conclusions of this manuscript will be made available by the corresponding author, without undue reservation, to any qualified researcher.

## Ethics Statement

The study was conducted according to the Helsinki Declaration II and the protocol was approved by the Ethics committees of Copenhagen Denmark. All subjects gave written informed consent in accordance with the declaration of Helsinki.

## Author Contributions

JB an HH designed the study. All authors participated in the study, discussed the results, and contributed to the final manuscript.

## Conflict of Interest Statement

The authors declare that the research was conducted in the absence of any commercial or financial relationships that could be construed as a potential conflict of interest.
